# An analysis of actors participating in the design and implementation of workplace breastfeeding interventions in Mexico using the NetMap analysis approach

**DOI:** 10.3389/fpubh.2023.1192600

**Published:** 2023-11-08

**Authors:** Kathrin Litwan, Vania Lara-Mejía, Teresa Chahine, Sonia Hernández-Cordero, Mireya Vilar-Compte, Rafael Pérez-Escamilla

**Affiliations:** ^1^Department of Social and Behavioral Sciences, Yale School of Public Health, Yale University, New Haven, CT, United States; ^2^Research Center for Equitable Development EQUIDE, Universidad Iberoamericana, Mexico City, Mexico; ^3^Yale School of Management, Yale University, New Haven, CT, United States; ^4^Department of Public Health, Montclair State University, Montclair, NJ, United States

**Keywords:** breastfeeding support, lactation support, lactation program, working mothers, workplace, social network analysis, NetMap analysis, Mexico

## Abstract

**Introduction:**

While breastfeeding is recognized as providing optimal nutrition for infants and toddlers, maternal employment is a commonly mentioned barrier to breastfeeding. The goal was to (a) identify key actors participating in the design and implementation of workplace breastfeeding interventions in Mexico, (b) understand the complexity of interactions between the actors, and (c) map the connections and influence between the actors when looking into networks of Advice, Command, Funding, and Information.

**Method:**

Following the NetMap methodology, a total of 11 semi-structured interviews with 12 interview partners from 10 organizations were conducted. Interview data were analyzed, and networks were analyzed and visualized, using a social network mapping software.

**Results:**

A total of 83 actors from five different actor groups were identified. Four networks were constructed along the four types of connections: Advice, Command, Funding, and Information. The actors were connected by 580 connections with 446 unique links. Based on various network statistics, the Mexican Institute of Social Security, the Mexican Secretary of Labor and Social Welfare, UNICEF, and the Mexican Secretary of Health were identified to be key actors.

**Conclusion:**

To increase the likelihood of success of workplace breastfeeding interventions, the role of the actors “Employers” and “Women” needs to expand. They should be actively involved in the decision-making process, together with the identified key actors. It is further recommended to re-introduce a national breastfeeding strategy for Mexico that includes policies for workplace breastfeeding interventions.

## 1. Introduction

Breastfeeding is recognized by authoritative bodies as providing optimal nutrition for infants and children for at least the first 2 years of age. The World Health Organization (WHO) and UNICEF recommend the initiation of breastfeeding within 1 h after birth, exclusive breastfeeding for the first 6 months and continued breastfeeding accompanied with the introduction of complementary food for at least the first 2 years of life (1). Breastfeeding provides short- and long-term health benefits to the breastfeeding mother as well as to the breastfed infant and child. Breastfeeding protects mothers against the risk for breast cancer, ovarian cancer, and type 2 diabetes, while breastfed children have a lower risk for morbidity and mortality from infectious diseases, increased intelligence scores and a risk reduction for overweight and potentially for type 2 diabetes later in life (2–4). As such, it is important for governments to invest in improving breastfeeding outcomes among their populations.

Maternal employment and return-to-work after childbirth are commonly mentioned barriers to breastfeeding (5, 6). At the time of writing, 649 million women in the world who work in the formal or informal economy do not have access to adequate maternity benefits (7). In Mexico, according to data from the 2018–2019 National Health and Nutrition Survey (ENSANUT), the prevalence of exclusive breastfeeding in children under 6 months was 28.3%, while women who reported to have a paid job showed an even lower prevalence of 23.2% for the same indicator (8). While the provision of lactation rooms and nursing breaks are considered to be low-cost interventions (9), workplace lactation interventions have been shown to be positively associated with higher breastfeeding rates and longer breastfeeding duration (10) as well as with reduced absenteeism and improved workplace performance, commitment and retention (11, 12). An increasing number of governments and employers are introducing measures to support working mothers in reaching their breastfeeding goals (13) which enables families to better combine their work responsibilities with their infant feeding goals. In order to support the compatibility of family and work responsibilities for more families and to support governments to reach the Sustainable Development Goals by 2030 (14), there is a need for strengthening current workplace interventions and for countries to introduce more robust guidelines and incentives to stimulate more employers to offer more workplace accommodations and support for nursing women.

To guide the scaling up of effective workplace breastfeeding interventions across different contexts, evidence-based policies are needed. One barrier in creating evidence-based policies that are tailored to different contexts is the involvement of local actors in the decision-making process. While there are different types of policy instruments (15, 16), there is a lack of evidence specifically informing decision making on different options across different contexts. To bring the right people to the table around such discussions, it is important to understand the key actors who are currently participating or who have the potential to engage in the design and implementation of sustainable workplace breastfeeding interventions in Mexico.

Breastfeeding interventions are complex service interventions (17) in which multiple actors are involved. Social network analysis methodologies can help to reach the goal of having a holistic understanding of the involved key actors and how they are connected and influence each other. This will be key for (a) selecting, co-designing and successfully implementing workplace breastfeeding support policies and interventions, (b) understanding the roles that different actors should have individually and as a complex network involving multidirectional interactions between actors, and (c) integrating multiple views into an effective consensus process to eventually agree on the choice of policies and corresponding interventions that are collectively endorsed by the network of actors.

The goals of this study were to (a) identify key actors participating in the design and implementation of successful workplace breastfeeding interventions in Mexico, (b) understand the complexity of interactions between the various actors in this area, and (c) map in detail how the actors are connected and influence each other across different networking dimensions including Advice, Command, Funding, and Information. The analysis of the actor networks will be conducted following the NetMap methodology as it allows to aggregate the expertise of multiple key informant partners into a common understanding of the field.

## 2. Methods

### 2.1. Ethical disclosure

The study was approved by the Ethics Committee from Universidad Iberoamericana Mexico City and received IRB exemption from the Institutional Review Board of Yale University. A signed written privacy statement was obtained from all interview participants.

### 2.2. Study design and approach

The main goals of the study were to identify key actors for designing and implementing successful workplace breastfeeding interventions for the Mexican context and to gain a holistic understanding of the interactions between the actors. To complete these goals, we conducted a stakeholder analysis using the NetMap methodology developed by the International Food and Policy Research Institute (IFPRI) (18, 19). It combines social network analysis and power mapping activity in a participatory interview technique. NetMap helps to understand and visualize the actor network at play and to identify key actors that are involved in a given area (19).

The NetMap interviews were structured in three steps: (1) *Actor mapping* – the goal of this step was to identify and visualize all possible key actors who are involved in successful workplace breastfeeding interventions in Mexico. This step was designed to answer the primary research question of “Who is involved in the successful design and implementation of workplace breastfeeding interventions in Mexico?” During this step, interview partners were asked to name all the actors that they could think of and assign them to one of the following four actor groups: government, non-governmental organization (NGO), academic or research institution, or other. During the Zoom interviews, actors were written in color-coded text boxes (according to the assigned actor group) or on color-coded self-adhesive paper notes for the in-person interviews. (2) *Linking actor networks* – the goal of this step was to understand and visualize the ways in which the actors are connected to one another. The interview partners were asked to connect the actors to one another, indicating the flow of power/influence between them. The flow could be identified as either unidirectional (i.e., from actor A to actor B but not from actor B to actor A) or bidirectional (i.e., from actor A to actor B and vice versa). Connections were color-coded based on the four types of connection presented to the interview partners (Table 1). (3) *Power mapping* – the goal of this last step was to identify and visualize the relative power/level of influence that actors on the map had over one another in relation to the successful design and implementation of workplace breastfeeding interventions in Mexico. To complete this last activity, interview partners were asked to assign relative power to each actor on a scale from 0 (this actor does not at all influence the success of workplace breastfeeding interventions in Mexico) to 5 (this actor influences the success of workplace breastfeeding interventions in Mexico).

**Table 1:** Types of connections between actors used to identify networks of actors participating in the design and implementation of workplace breastfeeding interventions in Mexico.

Type of connection	Description
Advice	Actors are linked by giving or receiving advice (e.g., one actor explains another actor how to do something best)
Command	Actors are linked by giving or receiving commands (e.g., one actor tells the other what to do)
Funding	Actors are linked by giving or receiving money or financial incentives (e.g., one actor funds a project of another actor)
Information	Actors are linked by giving or receiving information (e.g., one actor gives out information about something to another actor)

#### 2.2.1. Identification of interview partners

A preliminary list of potential interview partners was identified by two of the co-authors (SH-C and MV-C) based on the co-authors’ comprehensive knowledge of and engagement with breastfeeding policies in Mexico. The list included individuals representing government agencies, NGOs, academic organizations/research institutions, international organizations as well as business associations, all of which were expected to have knowledge on workplace breastfeeding interventions in Mexico. Informal invitations, including a short description of the study, were sent via email by the co-authors (VL-M or SH-C) to clarify general interest for participation. Invitees that expressed interest in interview participation received a formal invitation by email. After acceptance, the interview partners received a written privacy statement with the request to sign and send back to the research team prior to the interview. In total, 14 formal invitations to representatives of government agencies (*n* = 4), NGOs (*n* = 2), academic organizations/research institutions (*n* = 2), international organizations (*n* = 2) and others (*n* = 4) were sent. Of the 14 key informants invited, 12 agreed to be interviewed.

#### 2.2.2. Interviews

All but one of the interviews were conducted with one interviewee. One interview was conducted with two interview partners following the request of the participating organization given the complementary expertise of the two interview partners. This resulted in a total of 11 interviews with 12 interview partners. Of the 11 interviews, four interviews were conducted with representatives from governmental organizations, two interviews each were held with representatives from NGOs, academia/research institutions, and international organizations, respectively, and one interview was conducted with a representative of an organization classified as “other.” Table 2 gives an overview of the organizations represented during the interviews. The interviews were conducted in English (*n* = 6) or in Spanish (*n* = 5) depending on the preference of the interview partner. All but one of the interviews were conducted online using the Zoom platform. The one in-person interview was conducted in the office of the interviewee.

**Table 2:** Organizations represented by interviewees during the NetMap interviews to identify key actors participating in the design and implementation of workplace breastfeeding interventions in Mexico.

Actor group	Number of interviews	Number of interview partners	Organizations represented
Government	4	5	IMSS, STPS, CNEGSR, SIPINNA
NGO	2	2	Pacto por la Primera Infancia, ACCLAM
Academia/Research	2	2	IBERO, INSP
International organizations	2	2	UNICEF
Others	1	1	PALMA
Total	11	12	10

ACCLAM, Association of International Board Certified Lactation Consultants in Mexico; CNEGSR, National Center for Gender Equity and Reproductive Health; IBERO, Universidad Iberoamericana Mexico City; IMSS, Mexican Institute of Social Security; INSP, National Public Health Institute; PALMA, Proyecto de Apoyo a la Lactancia Materna; SIPINNA, National System for the Protection of Children and Adolescents; STPS, Secretariat of Labor and Social Welfare; UNICEF, United Nations Children’s Fund.

Interviews were led by a co-author (KL or VL-M) using a semi-structured interview guide developed for this study (Supplementary material). The other co-author (VL-M or KL) acted as note taker during the interviews. All interview materials were developed in English and then translated into Spanish. For the online interview, a Microsoft PowerPoint template (Supplementary Figure 1) was developed including color-coded text boxes for the actor mapping, color-coded arrows for the linking actor networks and pictograms of “power towers” for the power mapping activity. The Microsoft PowerPoint template was shared with the interview partner using the “share screen” function on the Zoom platform and remote control was given to the interviewees to populate the maps. For the in-person interview, a white board, color-coded self-adhesive paper notes for the actor mapping, white-board markers for the linking actor networks activity and “power tower” notes for the power mapping activity were used. Upon request of the interviewees, the guiding interview questions were shared with the interviewees prior to the interview.

During each interview, three maps – one per interview step (described earlier), were created and a picture of each of the maps was taken. All interviewees agreed for the interviews to be video and/or audio recorded. The interviews were conducted between October 13, 2022, and December 2, 2022, and lasted between 80 and 110 min, each.

### 2.3. Data management and analysis

Data on actor names, actor group allocation, assigned relative power as well as links between actors were entered in a separate Microsoft Excel sheet for each interview. Actor data (name and group allocation) were compared across all 11 interviews to ensure consistency. Any inconsistency in actor data was recoded based on the majority of responses across the interviews. During the recoding, a new actor group labeled “UN Organization” was added. Recoding was discussed among the co-authors until a consensus was reached.

Actor data from all 11 interviews were merged and the number of actor citations were reported from the combined data. A weighted average relative power for each actor based on the formula presented below was calculated (Eq. 1). For every interview j that did not mention actor i, we assigned a relative power of 0 to actor i for interview j. To reduce the background noise potentially created by actors cited only once, we excluded all single-cited actors from the analysis if the single-cited actor was assigned a relative power equal or less than 1 and was not part of any links among actors (*n* = 9, see Supplementary Table 1). This decision was made since it is unlikely that actors that were only cited by one interviewee, were assigned a low relative power and that were not part of any interaction will play an important role in the successful design and implementation of workplace breastfeeding interventions in Mexico.

Equation 1. Formula to calculate weighted average relative power for actor i across all j interviews.


$$\begin{array}{c} \text{Weighted}\;\text{average}\;\\ \text{relative}\;\text{power}\;\text{for}\;\text{actor}\;i \end{array}\;=\frac{\sum_{j=1}^{11}\;\text{Relative}\;\text{powe}r_{ij}}{11},\;\text{where}\;j\;\text{the}\;\text{interview}$$

Data from the linking actors network step from all 11 interviews were merged into one Microsoft Excel sheet for every type of connection (i.e., advice, command, funding, and information). Every link was weighted according to how many times the directed relation between the two actors was mentioned across the interviews (i.e., a relationship from one actors to another received a weight of 1 if it was mentioned in only one interview and a weight of 11 if it was mentioned in all 11 interviews). All merged data sheets were imported into the Gephi (version 0.10.0) network analysis software (20).

A network of actors was created for every type of connection (i.e., advice, command, funding, and information). Every network consists of actors represented by a node and links represented by arrows connecting two actors together. Nodes were color-coded based on their actor group (pink for governmental organizations, yellow for NGOs, green for academic/research institutions, blue for UN organizations and orange for “others”) and sized proportionally to their weighted average relative power. Larger nodes represent actors with higher weighted average relative power. The maps of actor networks are a representation of the summative views and experiences of the interview partners as actors and links are reported as stated by the interviewees. Each map was created by using the Yifan Hu algorithm (21). Following the Yifan Hu algorithm, minor cosmetic adjustments were undertaken to increase the readability of the maps: Overlapping nodes (actors) were moved such that they do not overlap in the final map, using the Gephi’s Noverlap plugin (22).

Network density and average degree as well as measures of centrality are used to describe the networks. While network density and average degree are network-level descriptive statistics and help to understand the network as a whole, measures of centrality are node-level descriptive statistics and help to understand the role of single actors within the network and compare actors within the same network. An overview of used network statistics, their definition and importance can be found in Table 3. In the following, we will report the actor distribution across actor groups (i.e., government agency, NGO, UN organization, university/research institution, and others), the number of times each actor was cited across all interviews as well as an evaluation across the four resulting networks.

**Table 3:** Definition and importance of statistics used to describe social networks (23, 24).

Statistics	Definition	Used for
Network-level		
Average degree	Average number of connections per node	To describe connectivity of network
Density	Percentage of all possible links that exist in the network	To describe connectivity of network
Node-level		
Degree/degree centrality	Number of connections a node has	To find very connected nodes
In-degree centrality	Number of incoming connections a node has	To find nodes that are largely receiver of a connection
Out-degree centrality	Number of outgoing connections a node has	To find nodes that are largely starting a connection
Betweenness centrality	Extend to which a node connects other nodes that are not otherwise connected	To find nodes that influence the flow around the system
Closeness centrality	Measure for how close a node is to all other nodes	To find nodes that are well placed to influence other nodes
Weighted average relative power	Average power an actor has to influence the success of workplace breastfeeding interventions in Mexico	To find influential actors

## 3. Results

### 3.1. Characteristics of actors

**Figure 1:**
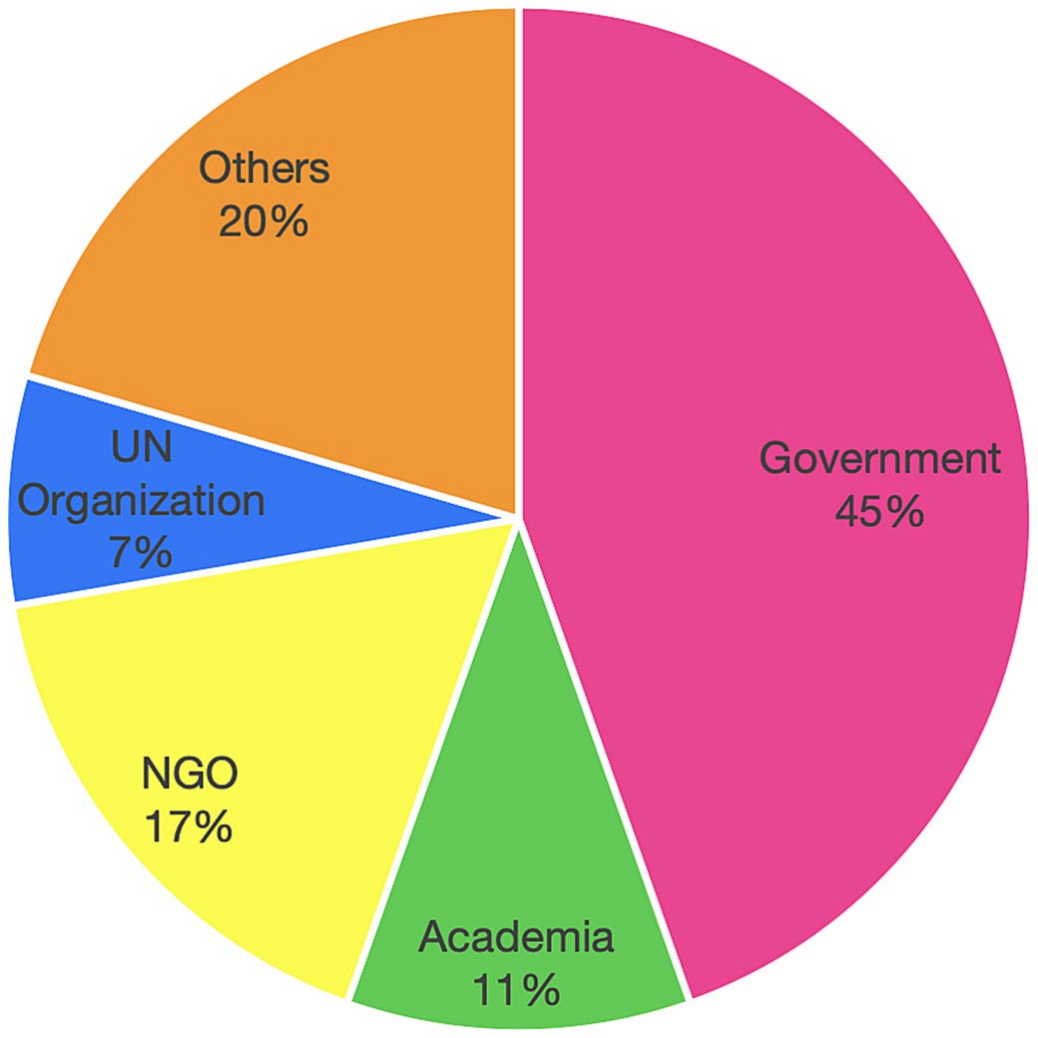
Distribution of actors participating in the design and implementation of workplace breastfeeding interventions in Mexico; based on actor groups.

After excluding single-cited actors with an assigned relative power of 0 or 1 and without any links in any of the networks, a total of 83 actors were included in the analysis. The majority of actors (*n* = 37) were from governmental organizations, followed by organizations labeled as “Others” (*n* = 17), NGOs (*n* = 14), academic or research institutions (*n* = 9) and UN organizations (*n* = 6; Figure 1). Examples of actors that were assigned to the actor group “Others” included “Employers,” “Business groups,” “Women,” “Private companies,” “Families and colleagues,” “Social media,” “Healthcare professionals,” and “Media.” The actor group of “UN Organization” was introduced after the researchers examined the interview data. Supplementary Table 2 lists all actors, their assigned actor group as well as the number of citations and the weighted average relative power.

**Figure 2:**
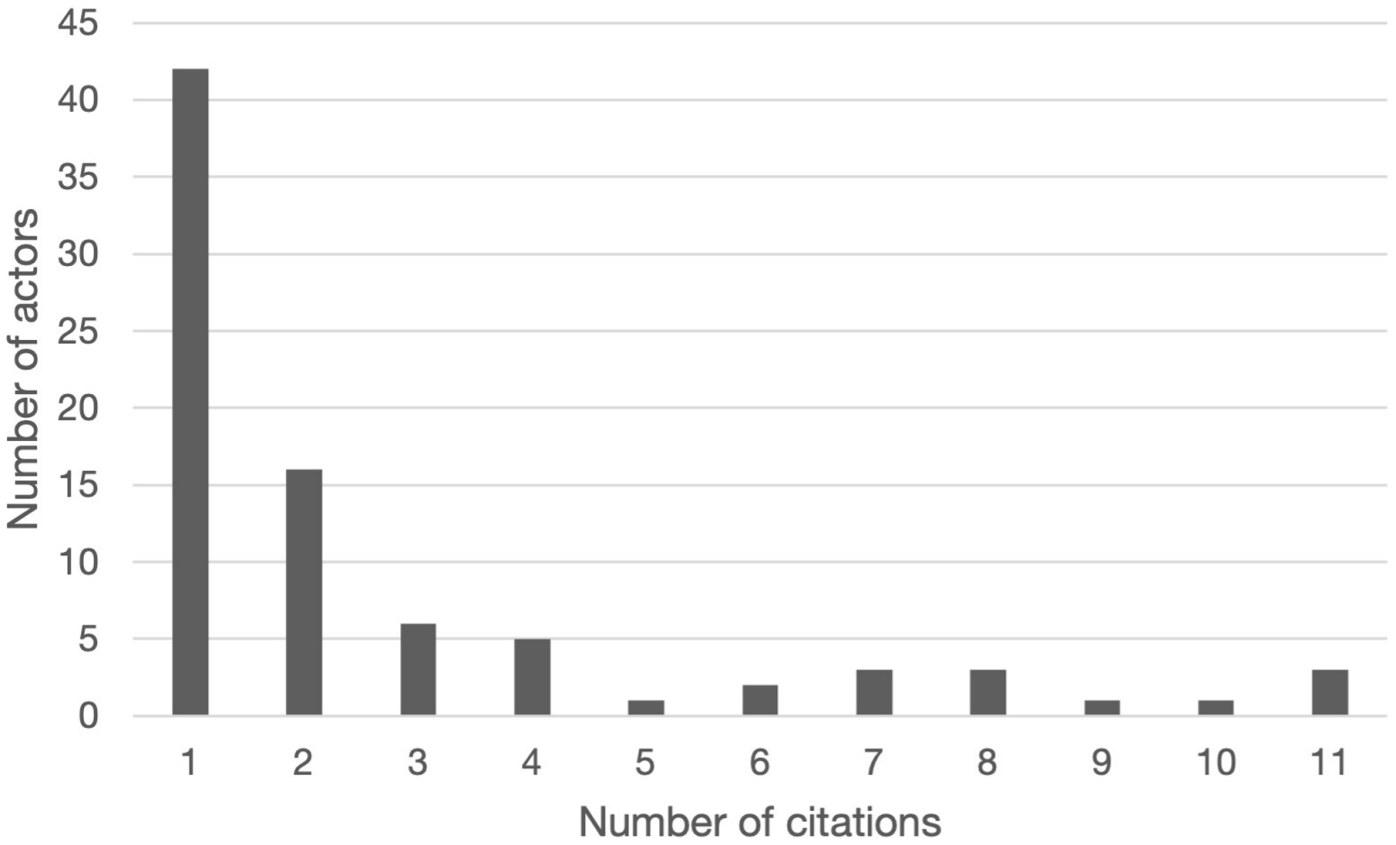
Frequency at which actors were mentioned during the 11 NetMap interviews to identify actors participating in the design and implementation of workplace breastfeeding interventions in Mexico.

Of the 83 actors, 42 actors were only mentioned in one interview (Figure 2). The Mexican Institute of Social Security (IMSS: Instituto Mexicano del Seguro Social), the Secretariat of Labor and Social Welfare (STPS: Secretaría del Trabajo y Previsión Social) and UNICEF were mentioned in all 11 interviews. The Universidad Iberoamericana Mexico City (IBERO) was mentioned in 10 of the 11 interviews, the Secretariat of Health (SALUD) was mentioned in 9 interviews, and the Association of International Board Certified Lactation Consultants in Mexico (ACCLAM: Asociación de Consultores Certificados en Lactancia Materna), the National Public Health Institute (INSP: Instituto Nacional de Salud Pública) and La Leche League were mentioned in 8 interviews.

**Figure 3:**
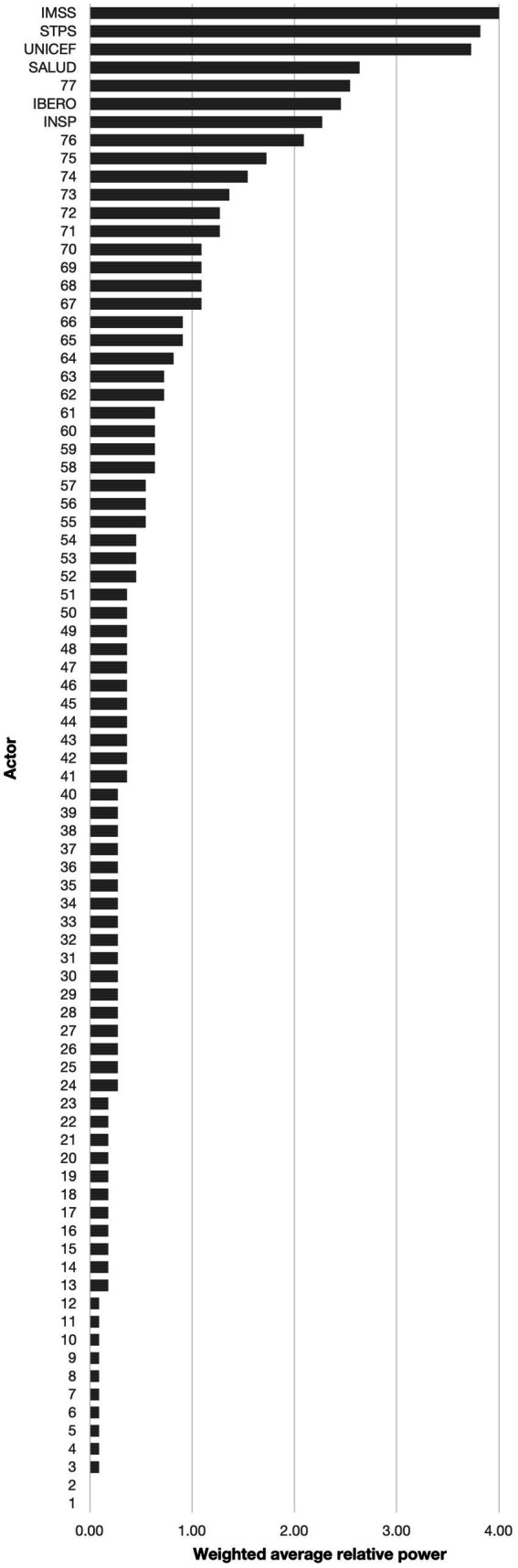
Weighted average relative power for all actors participating in the design and implementation of workplace breastfeeding interventions in Mexico; ranked from lowest to highest. 1: Media; 2: Labor unions; 3: National Council to Prevent Discrimination (CONAPRED); 4: Governor of the State of Sinaloa; 5: Infancia plena; 6: Secretariat of Labor and Employment Promotion; 7: State Secretariats of the Treasury and Public Credit; 8: Monterrey Institute of Technology and Higher Education; 9: University of Guadalajara; 10: UN Global Compact; 11: Autonomous University of the State of Hidalgo; 12: Volunatriodo de la Secretaría de Salud (Volunteer of the Ministry of Health); 13: Fundación DIANUI; 14: National Institute for Perinatology; 15: National Polytechnic Institute; 16: Mariana Villalobos; 17: Multi-stakeholder platforms (e.g., Centro Mexicano para la Filantropía); 18: Secretariat of Public Education (SEP); 19: State legislators; 20: Metropolitan Autonomous University; 21: State governments; 22: World Health Organization (WHO); 23: Private health sector; 24: Asociación Pro Lactancia Materna (APROLAM); 25: Chamber of Senators of the Honorable Congress of the Union; 26: National Center for Child and Adolescent Health (CeNSIA); 27: Child daycare centers; 28: Center for Economic and Budgetary Research (CIEP); 29: Parliamentary Front against Hunger (FPH) of the Chamber of Deputies of the General Congress of the United Mexican States; 30: IMSS-Bienestar; 31: Local offices of the Mexican Institute of Social Security; 32: Local offices of the National Institute for Women; 33: Punto de lactancia; 34: Save the Children; 35: Secretariat of Communication and Transportation; 36: Secretariat of Municipal Public Services; 37: Women’s NGOs (e.g., GIRE); 38: Un Kilo de Ayuda; 39: UN Women; 40: International Labor Organization (ILO); 41: Alianza por la Salud Alimentaria; 42: Committee on Children and Adolescent of the Chamber of Deputies; 43: Committee on Health of the Chamber of Deputies; 44: Committee on Social Security of the Chamber of Deputies; 45: Secretariat of Public Services; 46: Secretariat of National Defense; 47: Naval Secretariat; 48: Judiciary; 49: National System for the Protection of Children and Adolescents (SIPINNA); 50: National Autonomous University of Mexico (UNAM); 51: Doctors and Researchers in the Fight against Breast Cancer (milc); 52: Support groups of mothers; 53: Supreme Court of Mexico; 54: Social media; 55: Healthcare professionals; 56: Federal Commission for the Protection against Health Risks (COFEPRIS); 57: Proyecto de Apoyo a la Lactancia Materna (PALMA); 58: Corporate foundations; 59: PEMEX (Mexican state-owned petroleum company); 60: National Center for Gender Equity and Reproductive Health (CNEGSR); 61: National Institute for Women; 62: Secretariat of the Treasury and Public Credit; 63: Private companies; 64: State Secretariats of Labor and Social Welfare; 65: Families and colleagues; 66: Pan American Health Organization (PAHO); 67: Chamber of Deputies; 68: State Secretariats of Health; 69: Institute for Social Security and Services for State Workers (ISSSTE); 70: La Leche League; 71: Breastmilk substitute industry; 72: Association of International Board Certified Lactation Consultants in Mexico (ACCLAM); 73: Women; 74: Pacto por la Primera Infancia; 75: Business groups (e.g., chamber of commerce, COPARMEX); 76: Employers; 77: Federal legislators; IBERO: Universidad Iberoamericana Mexico City; IMSS: Mexican Institute of Social Security; INSP: National Public Health Institute; SALUD: Secretariat of Health; STPS: Secretariat of Labor and Social Welfare; UNICEF: United Nations Children’s Fund.

The weighted average relative power of actors ranged from 0.00 to 4.00 (Figure 3). IMSS (4.00), STPS (3.82), and UNICEF (3.73) were the actors with the highest weighted average relative power. Five actors (Employers, INSP, IBERO, Federal legislators and SALUD) had weighted average relative powers between 2.01 and 3.00 while 9 actors (Chamber of Deputies, State Secretariats for Health, Institute for Social Security and Services for State Workers, La Leche League, breastmilk substitute industry, ACCLAM, Women, Pacto por la Primera Infancia, and Business groups) had weighted average relative powers between 1.01 and 2.00. The majority of the actors (*n* = 66) had weighted average relative powers between 0.00 and 1.00 with 49 actors with a weighted average relative power between 0.09 and 0.39 and 2 actors (Media and Labor unions) with a weighted average relative power of 0.00 indicating that these actors were perceived by the interviewees as not influencing the success of workplace breastfeeding interventions in Mexico.

### 3.2. Networks of actors participating in workplace breastfeeding interventions in Mexico

All four networks are described below. Each network is depicted as a network map in which the actors that are participating in a respective connection are depicted as a node while the directed connections are shown as arrows going from the actor that initiates the relationship to the actor that is the receiving actor of this relationship. Table 4 gives an overview of the statistics of each of the four networks. Networks including their maps and statistics were developed as a result of the four types of connections between actors: Advice, Command, Funding and Information. Among the 83 actors, 4 actors (Labor unions, Chamber of Senators, Judiciary and the Supreme Court of Mexico) had no links with other actors. A total of 22 actors were part of all four networks. The number of actors participating in the network ranged from 34 in the Command network to 62 in the Advice network. The Information network had a total of 42 participating actors while 51 actors participated in the Funding network.

**Table 4:** Network statistics of actors participating in the design and implementation of workplace breastfeeding interventions in Mexico for the Advice, Command, Funding, and Information networks.

	Advice network	Command network	Funding network	Information network
Actors participating in links	62	34	51	42
Number of links (incl. multiple citations)	218	72	105	185
Number of unique links	168	51	84	143
Network density	0.025	0.007	0.012	0.021
Average degree	2.024	0.614	1.012	1.723
Average weighted degree	2.627	0.867	1.265	2.229
Betweenness centrality – Top 1 actor	UNICEF (492.95)	STPS (37.17)	UNICEF (144.00)	SALUD (446.36)
Betweenness centrality – Top 2 actor	SALUD (429.74)	IMSS (27.33)	SALUD (77.50)	UNICEF (286.10)
Betweenness centrality – Top 3 actor	IMSS (404.45)	SALUD (24.17)	Private companies (23.00)	STPS (198.05)
Weighted Degree – Top 1 actor (Centrality/weighted centrality)	UNICEF (28/51)	SALUD (16/24)	UNICEF (16/27)	SALUD (32/44)
Weighted Degree – Top 2 actor (Centrality/weighted centrality)	SALUD (30/44)	STPS (12/21)	Chamber of deputies (14/16)	STPS (24/35)
Weighted Degree – Top 3 actor (Centrality/weighted centrality)	IMSS (27/41)	IMSS (10/17)	SALUD (10/15)	IMSS (22/30), UNICEF (20/30)
Weighted In-Degree – Top 1 actor (Centrality/weighted centrality)	IMSS (18/31)	Employers (5/13)	INSP (5/10)	SALUD (20/30)
Weighted In-Degree – Top 2 actor (Centrality/weighted centrality)	Federal legislators (16/21)	IMSS (6/10)	IMSS (7/9)	STPS (15/23)
Weighted In-Degree – Top 3 actor (Centrality/weighted centrality)	STPS (14/20)	ISSSTE (4/6)	UNICEF (6/7)	Federal legislators (15/18)
Weighted Out-Degree – Top 1 actor (Centrality/weighted centrality)	UNICEF (22/44)	SALUD (14/22)	UNICEF (10/20)	UNICEF (14/20)
Weighted Out-Degree – Top 2 actor (Centrality/weighted centrality)	SALUD (21/26)	STPS (7/16)	Chamber of deputies (14/16)	IMSS (9/15)
Weighted Out-Degree – Top 3 actor (Centrality/weighted centrality)	ACCLAM (14/19)	Women’s NGO (e.g., GIRE) (7/7), IMSS (4/7)	BMS industry (9/11)	SALUD (12/14)

ACCLAM, Association of International Board Certified Lactation Consultants in Mexico; BMS industry, breastmilk substitute industry; IMSS, Mexican Institute of Social Security; INSP, National Public Health Institute; ISSSTE, Institute for Social Security and Services for State Workers; SALUD, Secretariat of Health; STPS, Secretariat of Labor and Social Welfare; UNICEF, United Nations Children’s Fund.

The interview partners mentioned a total of 580 connections. Excluding all multiple mentioning of connections, a total of 446 links across all type of connections could be counted (referred to as “unique links”). The Advice network had the highest number of unique links (*n* = 168) followed by the Information network (*n* = 143), the Funding network (*n* = 84) and the Command network (*n* = 51).

#### 3.2.1. Advice network

**Figure 4:**
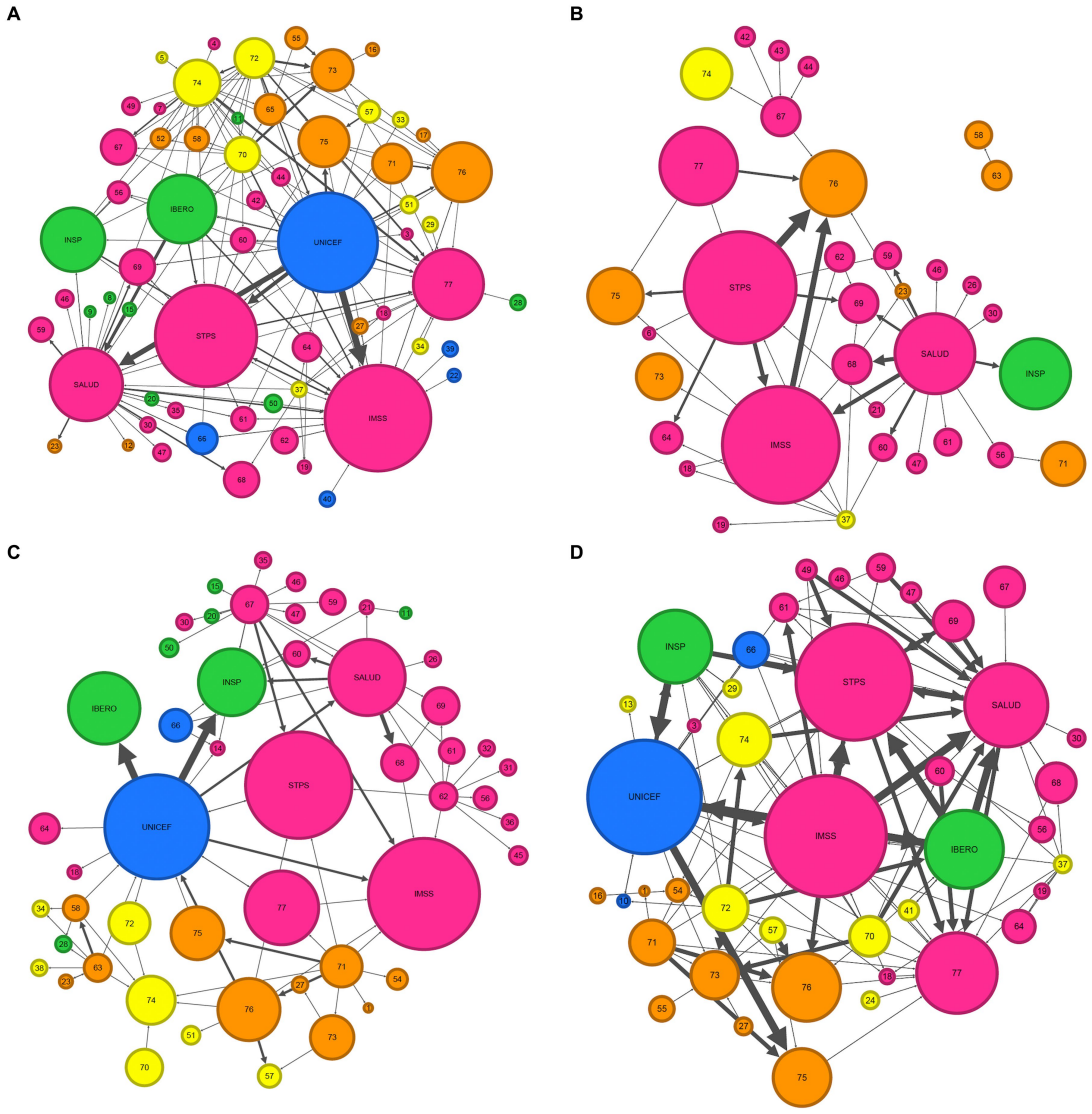
Maps of the Advice **(A)**, Command **(B)**, Funding **(C)**, and Information **(D)** networks of actors participating in the design and implementation of workplace breastfeeding interventions in Mexico. Actor group colors: pink = government, yellow = NGO, green = academia/research institution, blue = UN organization, orange = others. Arrows: Arrows indicate the direction of relationship, e.g., the direction of advice [which actor is providing advice to which actor in the Advice network **(A)**]. The thickness of the arrow represents the number of citations this relationship has been mentioned during the NetMap interviews, i.e., the weight of the link, and thus, the robustness of the relationship with thinner arrows only having a single citation and thicker arrows representing connections that have been cited multiple times across the NetMap interviews. Actors: 1: Media; 3: National Council to Prevent Discrimination (CONAPRED); 4: Governor of the State of Sinaloa; 5: Infancia plena; 6: Secretariat of Labor and Employment Promotion; 7: State Secretariats of the Treasury and Public Credit; 8: Monterrey Institute of Technology and Higher Education; 9: University of Guadalajara; 10: UN Global Compact; 11: Autonomous University of the State of Hidalgo; 12: Volunatriodo de la Secretaría de Salud (Volunteer of the Ministry of Health); 13: Fundación DIANUI; 14: National Institute for Perinatology; 15: National Polytechnic Institute; 16: Mariana Villalobos; 17: Multi-stakeholder platforms (e.g., Centro Mexicano para la Filantropía); 18: Secretariat of Public Education (SEP); 19: State legislators; 20: Metropolitan Autonomous University; 21: State governments; 22: World Health Organization (WHO); 23: Private health sector; 24: Asociación Pro Lactancia Materna (APROLAM); 26: National Center for Child and Adolescent Health (CeNSIA); 27: Child daycare centers: 28: Center for Economic and Budgetary Research (CIEP); 29: Parliamentary Front against Hunger (FPH) of the Chamber of Deputies of the General Congress of the United Mexican States; 30: IMSS-Bienestar; 31: Local offices of the Mexican Institute of Social Security; 32: Local offices of the National Institute for Women; 33: Punto de lactancia; 34: Save the Children; 35: Secretariat of Communication and Transportation; 36: Secretariat of Municipal Public Services; 37: Women’s NGOs (e.g., GIRE); 38: Un Kilo de Ayuda; 39: UN Women; 40: International Labor Organization (ILO); 41:Alianza por la Salud Alimentaria; 42: Committee on Children and Adolescent of the Chamber of Deputies; 43: Committee on Health of the Chamber of Deputies; 44: Committee on Social Security of the Chamber of Deputies; 45: Secretariat of Public Services; 46: Secretariat of National Defense; 47: Naval Secretariat; 49: National System for the Protection of Children and Adolescents (SIPINNA); 50: National Autonomous University of Mexico (UNAM); 51: Doctors and Researchers in the Fight against Breast Cancer (milc); 52: Support groups of mothers; 54: Social media; 55: Healthcare professionals; 56: Federal Commission for the Protection against Health Risks (COFEPRIS); 57: Proyecto de Apoyo a la Lactancia Materna (PALMA); 58: Corporate foundations; 59: PEMEX (Mexican state-owned petroleum company); 60: National Center for Gender Equity and Reproductive Health (CNEGSR); 61: National Institute for Women; 62: Secretariat of the Treasury and Public Credit; 63: Private companies; 64: State Secretariats of Labor and Social Welfare; 65: Families and colleagues; 66: Pan American Health Organization (PAHO); 67: Chamber of Deputies; 68: State Secretariats of Health; 69: Institute for Social Security and Services for State Workers (ISSSTE); 70: La Leche League; 71: Breastmilk substitute industry; 72: Association of International Board Certified Lactation Consultants in Mexico (ACCLAM); 73: Women; 74: Pacto por la Primera Infancia; 75: Business groups (e.g., chamber of commerce, COPARMEX); 76: Employers; 77: Federal legislators; IBERO: Universidad Iberoamericana Mexico City; IMSS: Mexican Institute of Social Security; INSP: National Public Health Institute; SALUD: Secretariat of Health; STPS: Secretariat of Labor and Social Welfare; UNICEF: United Nations Children’s Fund.

Actors that exchange advice about workplace breastfeeding interventions are described in the Advice network (Figure 4A). The Advice network was the biggest network with 62 actors and 168 unique links. Four out of 10 actors with links in this network were governmental organizations (40.32%) and 20.79% were from “other” organizations. The network density was 0.025, thus 2.5% of all possible links between all actors in the network had been achieved. The average distance between any two actors in the network was 2.70. UNICEF was the actor with the greatest betweenness centrality (492.95) suggesting an important role in connecting other actors in the network to each other. UNICEF was also the actor with the highest number of links (weighted degree = 51) and who provided most of the advice to the other actors (weighted out-degree centrality = 44). Of the 22 unique links from UNICEF to other actors, 14 links were going to governmental actors. IMSS was the actor that received most of the advice (weighted in-degree centrality = 31). Of the total 18 incoming unique links to IMSS, six links came from a UN Organization and five links came from a governmental actor.

#### 3.2.2. Command network

The Command network (Figure 4B) describes actors that provide or receive command regarding workplace breastfeeding interventions. It was the smallest network with 34 participating actors and 51 unique links. Governmental organizations made 70.59% of participating actors followed by actors grouped as “others” (20.59%). There were no actors from UN Organizations participating in the Command network. The network density of the Command network was 0.007 and the average distance between any two actors was 1.75. With a betweenness centrality of 37.17, STPS was the most central actor in the network. SALUD was the actor with the largest weighted degree (weighted degree = 24) and the largest number of out-going links (weighted out-degree centrality = 22). Of the 14 outgoing unique links from SALUD to other actors, 11 links were going to other governmental actors. Employers were the actor with the highest weighted in-degree centrality (weighted in-degree centrality = 13). All incoming links came from governmental actors. There was one isolated group of two actors that was not connected to the other actors in the network: Private companies provided command to Corporate foundations. Neither of these two actors was connected to any of the actors that were linked in the Command network.

#### 3.2.3. Funding network

Actors that provide or receive funding for the design or implementation of workplace breastfeeding interventions are included in the Funding network (Figure 4C). It consisted of 51 participating actors and had 84 unique links. Of the 51 participating actors, 49.02% were categorized as governmental organizations and 19.61% as “others.” The actor groups “Academic and research institutions” and “NGO” represented 13.73% of participating actors. The network density of the Funding network was 0.012 and the average distance between any two actors was 2.16. The actor with the highest betweenness centrality was UNICEF (betweenness centrality = 144.00). UNICEF was also the actor with the highest weighted degree (weighted degree centrality = 27.00) and weighted out-degree centrality (weighted out-degree centrality = 20.00). Of the 10 outgoing unique links from UNICEF, 7 links were going to governmental actors. The actor with the largest weighted in-degree centrality was INSP (weighted in-degree centrality = 11.00). Of the 5 incoming unique links to INSP, 3 were coming from governmental actors and 2 were coming from UN Organizations.

#### 3.2.4. Information network

The Information network (Figure 4D) includes all the actors that are providing or receiving information about workplace breastfeeding interventions. It had 42 participating actors and 143 unique links. Of all participating actors, 45.24% were governmental actors, followed by NGO and “other” actors with each 21.43%. The network density of the Information network was 0.021 and the average distance between any two actors was 2.84. SALUD had the greatest betweenness centrality (446.36) indicating that SALUD is very central in the network. The highest out-degree centrality had UNICEF (weighted out-degree centrality = 20.00). Of the 13 unique links going from UNICEF to other actors, seven links were going to governmental actors. SALUD was the actor with the highest weighted in-degree centrality (weighted in-degree centrality = 30.00). Of the 20 incoming unique links to SALUD, 13 were coming from governmental organizations.

The interview data allowed us to describe the field of actors participating in the design and implementation of workplace breastfeeding interventions in Mexico in four networks: the Advice network, Command network, Funding network and Information network. Each of the networks describe how the different actors in the field are connected to each other based on the type of relationship they share. In all four networks, the majority of actors belonged to the actor group of “governmental organization” indicating the important role of the government in designing and implementing workplace breastfeeding interventions in Mexico. Based on the network statistics and given their position in the different networks, IMSS, STPS, UNICEF, and SALUD were identified as key actors in the design and implementation of workplace breastfeeding interventions in Mexico with UNICEF being the only non-governmental actor in this group of key actors.

## 4. Discussion

This is the first study to identify the key actors for designing and implementing successful workplace breastfeeding interventions in the Mexican context. Out of the 83 actors from governmental, academic/research, NGO, UN or “other” organizations, we identified patterns in the top actors and the relationships between them. The actors IMSS (the Mexican Institute of Social Security), STPS (the Mexican Secretariat of Labor and Social Welfare), UNICEF, and SALUD (the Mexican Secretariat of Health) consistently emerged as the top actors with respect to different centrality measurements in the four analyzed networks of Advice, Command, Funding, and Information. This indicates that these four players hold an important role in the design and the implementation of successful workplace breastfeeding interventions in Mexico. More generally, our analysis identified that governmental actors were perceived by the interview partners to play an important role in the design and implementation of workplace breastfeeding interventions in Mexico. This perceived importance of governmental actors was further supported by network centrality measures and weighted average relative power. Besides always ranking among the top actors in means of centrality measures and weighted average relative power in each network, connections to and from governmental actors were also responsible for the high degree centrality of top actors in the respective networks. This suggests that governmental actors such as the IMSS, STPS and SALUD together with UNICEF need to be included in initiatives to change workplace breastfeeding interventions and policies in Mexico.

We want to highlight several points. The current analysis adds knowledge to a growing body of literature discussing social networks in the field of breastfeeding and more generally in the field of infant and young child feeding such as a previous NetMap analyses of breastfeeding policy and programming in Mexico (25), infant and young child feeding in India (26), and breastfeeding policies and programs in Ghana (27). Compared to a previous NetMap in Mexico (25), the current analysis identified a much larger number of actors in the field. This is remarkable as the analysis by Buccini and colleagues is a description of actors participating in the field of general breastfeeding policy and programming in Mexico rather than focusing on workplace breastfeeding interventions as this analysis has done. It is reasonable to assume that the number of participating actors would decrease when going from the general to a more specific perspective. As a consequence, this indicates that when discussing the design and implementation of workplace breastfeeding interventions in Mexico, more actors need to be included in the discussion which leads to the need for good coordination in order to be effective. Actors well positioned to take over the lead of such conversations are actors with a high centrality and a high relative power. Of the 83 identified actors, only three actors had a weighted average relative power above 3.50 while the remaining actors all showed a weighted average relative power below 3.00 indicating that IMSS, STPS and UNICEF are the most influential actors in the field, and thus, need to be included when discussing workplace breastfeeding interventions and policies in Mexico. Comparisons to other NetMap analyses in the fields of breastfeeding and infant and young child feeding vary. While the previous NetMap analysis in Mexico by Buccini et al. (25) and the NetMap analysis for infant and young child feeding in India (26) identified more actors with higher relative power, the NetMap analysis for breastfeeding policies and programs in Ghana (27) also identified only a small number of actors with a high relative power. Comparisons across different analyses is always difficult as the result of each analysis highly depends on contextual factors present at the time of the analysis. The fact that the current analysis presented only three actors with a middle range weighted average relative power (possible range went from 0.00 indicating that the actor does not at all influence the success of workplace breastfeeding interventions in Mexico to 5.00 indicating that the respective actor highly influences the success of such interventions) indicates that those three actors need to be at the table when workplace breastfeeding interventions are discussed but it also indicates that those actors have the opportunity to even strengthen their influence on the success of workplace breastfeeding interventions in Mexico by strengthening their focus, and thus, strengthening the entire field.

Governmental actors along with UNICEF were identified as most influential actors in the design and implementation of workplace breastfeeding interventions in Mexico throughout the different dimensions of Advice, Command, Funding, and Information. UNICEF was identified to be best positioned to coordinate advice and funding between the different actors while STPS was identified to be best positioned to coordinate command and SALUD had a central position to coordinate information among the participating actors. Among all four networks, there were only two actors not belonging to governmental agencies that had top-3 betweenness centrality measures in the respective networks: UNICEF and Private companies. The importance of governmental actors can also be seen, when looking at the breakdown of links of actors with highest degree measurements. Connections from or to governmental organizations were the main driver for the high degree centrality measures of top actors. Other studies also identified governmental actors as important for policies and programing of breastfeeding and more in general infant and young child feeding (25–28). While SALUD emerged to be the most central actor among all four networks in the analysis by Buccini et al. (25), SALUD was only best positioned to coordinate the flow of information between the actors in the current analysis. It is important to mention that the analysis by Buccini and colleagues focused on general breastfeeding policies and programing while the presented analysis focused especially on workplace breastfeeding interventions. It is therefore reasonable that other players such as STPS are attributed an important position in the field. But the lost importance of SALUD is likely also a result of political changes in Mexico that happened between the two analyses. Between 2006 and 2018, the Mexican government invested in a national breastfeeding strategy to promote, protect and support breastfeeding, thus, providing funding for respective initiatives. The current administration that is in office since 2018 did not continue the political and financial commitment from its previous administrations (29), thus forcing governmental actors to open up the space for other actors, in particular UNICEF. While governmental actors might have lost some of their previous importance in promoting, protecting, and supporting breastfeeding through respective interventions, this study shows that they are still important and need to be included in any discussions about the topic. As such, it is important to remind that governmental organizations need to work on the dissemination of and announcements about working mother’s rights to breastfeed after their return to work (30). Given the wide variety of backgrounds and expertise of our interview partners, we concluded that the sum of interviews will level out any potentially biased results and that the prominent appearance of governmental actors is a true result rather than an artifact, and thus, that governmental actors need to have a seat at the table when discussing and coordinating the design and implementation of workplace breastfeeding interventions in Mexico.

While the key role of governmental actors was to be expected to some extent, the authors were surprised by the relatively small role the actors “Employers” and “Women” played. As the implementer of any workplace breastfeeding policy, employers are a key actor, and their buy-in is critical to achieve the desired outcome of an environment that allows women to feel safe and comfortable making breastfeeding choices. Women are the ultimate end user of the policy, and their relatively small role in our results indicates a top-down approach that does not include the end user in formulating workplace breastfeeding interventions in Mexico. While the actor “Employers” was mentioned in 7 out of the 11 interviews with a weighted average relative power of 2.09, the actor “Women” was only mentioned in 3 interviews and received a weighted average relative power of 1.36. The relative low importance of the actors “Employers” and “Women” can also be seen when comparing their weighted average relative powers to the weighted average relative power of IMSS (4.00): The actor “Employers” has only about half of IMSS’ influence on the success of workplace breastfeeding interventions while the actor “Women” only has about a third of IMSS’ influence. It is possible that the way the questions during the interviews were framed gave rise to this low representation of “end users” of workplace breastfeeding interventions. The emphasis of “successful design and implementation” of workplace breastfeeding interventions could have been one reason interview partners did not immediately think of “Employers” and “Women” as important actors in the field. To ensure an uptake of workplace breastfeeding interventions and regardless of the result of the present analysis, it is critical that discussions around the design and implementation of workplace breastfeeding interventions center on women and their employers.

Low network densities are consistently reported in social network analyses in the field of breastfeeding and infant and young child feeding (25, 27, 31). Nevertheless, and when compared to the only NetMap analysis available for breastfeeding interventions in Mexico, network densities resulting from the present analysis seem to be particularly low. Compared to the previous NetMap analysis of breastfeeding policy and programming in Mexico by Buccini et al. (25), there was a notable drop in network density. Reasons for this drop can be manifold and a direct comparison between the two studies is to be taken with care. First of all, our analysis focused on the system that gives rise to workplace breastfeeding interventions while the analysis by Buccini et al. analyzed the more general breastfeeding governance system in Mexico not solely focusing on workplace breastfeeding interventions. Secondly, there are 5 years between the two analyses. The analysis by Buccini and colleagues’ is based on interviews conducted between November and December 2017 while the interviews for the present analysis were conducted between October and December 2022. In December 2018, and thus in between the two analyses, the current administration came into office. As previously mentioned, investments into national breastfeeding policies and programs that were initiated and supported by the administrations between 2006 and 2018 were not anymore supported by the new administration (29). It is further to mention, that the low density measurements are likely to be a result of chosen methodologies. We used the full list of all 83 actors for all four different networks instead of only using the list of participating actors per network, i.e., a list of actors that had connections to other actors in the respective network. This increased the denominator to calculate the percentage of all possible links within a network thus decreasing the resulting density measurements as well as the average degree measures. It is to mention that a network density (i.e., the percentage of all possible links that exist in a network) towards 1 is also not desirable as it seems very inefficient if everyone is connected to everyone (27). Furthermore, networks with lower connectivity (indicated by a low network density) also provide an opportunity for actors within the network to take the lead in connecting other actors and leading the development of the field. The identified key actors IMSS, STPS, UNICEF, and SALUD are in the prime position to take over the lead in strategically designing and implementing workplace breastfeeding interventions in Mexico by including the actors “Employers” and “Women.”

The previously mentioned decision of the current administration in Mexico to discontinue its commitments in a national breastfeeding strategy (29) can be understood as the contextual factor that results in the presented network characteristics. The high number of actors together with a high percentage of single-time actor citations and high proportion of unique links in relation to the total number of links are an indication for low agreement in the field about participating actors. The sudden removal of a national breastfeeding strategy is likely to have led to a disorganization of the field which can lead to a low agreement about actors in play. The previously discussed low network densities identified in this analysis and the seemingly low agreement among interviewees on participating actors lead to the conclusion that the design and implementation of workplace breastfeeding interventions is unstructured. Thus, in order to increase the efficiency and the success of workplace breastfeeding interventions, it is strongly recommended to re-introduce a national breastfeeding strategy for Mexico that includes policies for workplace breastfeeding interventions.

The analysis showed that perceived actor influence as rated by the interview partners was congruent with network statistics indicating relevance of actors. Each interview partner assigned a relative power (on a scale from 0 to 5, with 0 indicating that the actor does not at all influence the success of workplace breastfeeding interventions in Mexico and 5 indicating that the actor influences the success of workplace breastfeeding interventions in Mexico). The actors with the highest weighted average relative power IMSS (4.00), STPS (3.82), UNICEF (3.73), and SALUD (2.64) are the actors that were identified as the most influential actors based on different network statistics such as betweenness centrality and degree centrality. Furthermore, most of the top-10 actors based on weighted average relative power are also among the top-3 actors when looking at network centrality measures. Thus, despite that relative power is a measurement of influence perceived by the single interviewees, its aggregated form of weighted average relative power is a good first estimation of actor’s influence in the field in situations where there are no resources to conduct a full social network analysis.

To our knowledge, this analysis is the first published study using the NetMap methodology with a combination of online and in-person interviews. Based on personal preferences and time availability of our interview partners, the interview partners could choose between in-person and online interviews. We could not find any difference between the two interview types. There was no difference in the number of mentioned actors (23 actors were mentioned in the in-person interview vs. a median of 22 actors in the online interviews, data not shown) as well as in the number of mentioned relationships. Thus, our study could show that using online interviews instead of in-person interviews during the NetMap process is a valid alternative.

Besides being the first social network analysis of workplace breastfeeding interventions in Mexico with a clear identification of key actors following a robust study methodology and methodological contributions, our study is not without limitations. While our interview partners had a diverse background, all our interviewees worked on the national level rather than on the local level. Thus, our analysis does not allow us to identify local key actors for designing and implementing workplace breastfeeding interventions in Mexico. Furthermore, we conducted the interviews mostly with one representative of the respective organization. Only UNICEF and the National Center for Equity and Reproductive Health were represented by two interviewees. In both cases, the expertise of the interview partners was different between the two interviewees such that the additional representative added a second perspective while being associated with the same organization. In addition, we interviewed a relatively small number of participants, although they did identify a large number of actors. Given that the top ranked actors (UNICEF, IMSS, STPS, and SALUD) were mentioned in most of the interviews and mostly have been assigned similar relative powers across the interviews, we concluded that we reached saturation of information after the 11 conducted interviews. However, we acknowledge that there is the possibility that interviewing more than one representative from each organization and interviewing more interview partners in general could have led to more insights. But by consistently identifying the same actors as key actors, we feel comfortable that we did not miss an important actor that is participating in the design and implementation of workplace breastfeeding interventions in Mexico. Furthermore, even though we feel confident in our results we cannot rule out the introduction of two potential biases in our study. First, participants’ selection bias may have been present as the preliminary list of possible interview partners was identified through the co-authors’ networks in topics related to breastfeeding. Second, a bias may have been introduced because the relative power attributed to each actor was based on the subjective assessment of each interviewee. Those possible biases could only have been eliminated by expanding the number of interview participants which would have exceeded the scope of the study. We would also like to mention that the interview questions allowed us to identify actors that are currently participating in the successful design and implementation of workplace breastfeeding interventions but did not allow us to identify potential actors that currently do not participate but have the potential to do so. While the questions did not prompt the interview partners to actively think about actors that should, and have the potential, to play a role, the method of network analysis allows to at least identify potential for identified actors to adapt their roles. For example, the betweenness centrality of the identified key actors UNICEF, IMSS, STPS, and SALUD shows their potential to strengthen the field by fostering further connections between additional players. The actors “Employers” and “Women” have the potential to be such additional players. While the analysis did not reveal the actors “Employers” and “Women” as actors of high influence, we made the case that those two actors have the potential to, and thus, should be included to strengthen the success of workplace breastfeeding interventions. One possibility why we were not able to identify “Employers” and “Women” as actors of high influence is the fact that we did not have representatives of those two actor groups as interview partners. Another possibility is that until now they have not really played an influential role in setting or implementing workplace breastfeeding policies and programs. We recommend that research discussing the design of workplace breastfeeding interventions should include working mothers as well as employers for example by following the human-centered design approach (32, 33). Lastly, we would like to acknowledge that our study is a pure analysis of the system of actors. To be able to structure priorities in the field, it is not enough to only know the actors. Rather the entire system needs to be evaluated (34). So far and to the knowledge of the authors, there is currently no system analysis about the design and implementation of workplace breastfeeding interventions in Mexico available. Thus, and in order to strengthen the national efforts to support parents in reaching their breastfeeding goals, we would recommend conducting such a system analysis for workplace breastfeeding interventions in the Mexican context.

In order to best support working parents in reaching their breastfeeding goals at the national level, there needs to be clarity about who needs to be involved and about the choice of policy instruments. This analysis provides an overview of actors that participate in some capacity in the design and the implementation of workplace breastfeeding interventions in Mexico. It therefore can serve as a starting platform to discuss the best instruments or mix of instruments with the most important actors. Workplace breastfeeding policies in Mexico are currently mainly supported by regulatory instruments (the Mexican constitution as well as the Mexican labor law defines the women’s right for two 30-min extra breaks a day to nurse their infants during the first 6 months of life (35)). Discussions with involved actors need to involve discussions about other possible instruments such as economic and financial instruments as incentives for employers to implement workplace breastfeeding interventions, e.g., tax exemptions or subsidies for employers implementing workplace breastfeeding interventions. By discussing the best mix of interventions, the actors should also always remember that the interventions need to be flexible enough to be adapted to the context in which the intervention will be implemented (17). Families will be most supported if knowledge about workplace breastfeeding interventions as well as knowledge about policy instruments is applied in combination.

In conclusion, using the NetMap methodology, we identified IMSS (the Mexican Institute of Social Security), STPS (the Mexican Secretary of Labor and Social Welfare), UNICEF, and SALUD (the Mexican Secretary of Health) as key actors in designing and implementing workplace breastfeeding interventions in Mexico when looking at Advice, Command, Funding and Information relationships between actors. Our analysis also showed that besides these four key actors, in general governmental organizations played an important role. Furthermore, we laid out why the actors “Employers” and “Women” should also be included in future discussions around workplace breastfeeding interventions. The high number of actors together with a high number of unique relationships between actors were an indication for a fairly fragmented field. This bears the opportunity for interested actors to take over the lead to structure and develop the design and implementation of workplace breastfeeding interventions in Mexico. Therefore, findings from this analysis should be used as a starting point for directed discussions with actors that are positioned best to address policy recommendations in order to reach the best possible results for working mothers and their families.

## Data availability statement

The datasets presented in this article are not readily available because the confidentiality of the participating interview partners needs to be protected. Requests to access the datasets should be directed to KL, kathrin.litwan@yale.edu.

## Ethics statement

The studies involving humans were approved by the Ethics Committee from Universidad Iberoamericana Mexico City and received IRB exemption from the Institutional Review Board of Yale University. The studies were conducted in accordance with the local legislation and institutional requirements.

## Author contributions

KL designed the study with the help and input from SH-C, MV-C, and RP-E. KL led the development of the data collection tool and designed and conducted the data analysis with contributions from SH-C, MV-C, and RP-E. KL led the English NetMap interviews. VL-M led the Spanish NetMap interviews. VL-M led the interview administration under the supervision of KL and SH-C. KL led the development of the manuscript with input from VL-M, TC, SH-C, MV-C, and RP-E. All authors contributed to the article and approved the submitted version.
