# Supplementary material for: An analysis of actors participating in the design and implementation of workplace breastfeeding interventions in Mexico using the NetMap analysis approach
Source: Front Public Health. 2023 Nov 8;11:1192600. doi: 10.3389/fpubh.2023.1192600 (PMC10663280; doi:10.3389/fpubh.2023.1192600)
Supplement: Supplementary file 1 [file Table_1.PDF]

Supplementary Material

An Analysis of Actors Participating in the Design and Implementation of Workplace Breastfeeding Interventions in Mexico Using the NetMap Analysis Approach

Kathrin Litwan*, Vania Lara-Mejía, Teresa Chahine, Sonia Hernández-Cordero, Mireya Vilar-Compte, Rafael Pérez-Escamilla

*** Correspondence:** Kathrin Litwan: kathrin.litwan@yale.edu

1 NetMap interview guide for the identification of actors participating in the design and implementation of workplace breastfeeding interventions in Mexico.

Comment: The following interview guide was developed for the in-person interviews. It was adapted accordingly for the online interviews.

Present: Interviewee, interviewer, and note taker

Actor Mapping

[Use colored post-its]

The first step of our interview involves Actor Mapping. The goal of this step is to identify and visualize all possible key actors who are involved in successful workplace breastfeeding interventions in Mexico.

1. Who is involved in the successful design and implementation of workplace breastfeeding interventions in Mexico? That is, what are local, regional, and national actors that play a part in the design and implementation of successful workplace breastfeeding interventions in Mexico? Please write the name of each actor on a post-it. Choose the color that you think best describes the type of organization the actor belongs to, i.e., pink for a governmental organization, yellow for a non-governmental organization (NGO), green for a university or a research institution, and orange for other type of actors. Please list as many actors as you can think of. [Allow time to write actors on post-its.]
   Please arrange your post-its on this [poster paper/white board/…], placing the most powerful/influential actors in the middle of the map, and the others outside of those, accordingly to their level of influence. [Note: At this time, the note taker should write the names of the actors separately, noting if any actors outside of the previously identified sectors arise].
   a. Now that we have arranged all the post-its, please tell me: Who can influence successful workplace breastfeeding interventions in Mexico? [Refer to actors and their placement on the map, asking questions accordingly, such as "I noticed you placed X in the middle. Why did you do that?"]

[The note taker takes a photo of the actor map.]

Linking Actor Networks

[Drawing of arrows and addition of abbreviations]

The second step of our interview involves Linking Actor networks. The goal of this step is to be able to understand and visualize the ways in which the actors are connected to one another.

Actors can be linked to one another in many ways. We have identified some of the ways for you; if you have any other suggestions besides those ways that I will define, please let us know.

[Offer the following 4 domains of power/influence to the interviewee to define the type of link and add abbreviations close to the arrow. Present the interviewee with a printed-out list of these types of linkages.]

- **Command (C)**: actors are linked by giving or receiving commands (for example, one actor tells the other that it must do something)
- **Funding (F)**: actors are linked by giving or receiving money or financial incentives (for example, one actor funds projects within another one)
- **Advice (A)**: actors are linked by giving or receiving advice (for example, one actor explains how another actor would best do something)
- **Information (I)**: actors are linked by giving or receiving information (for example, one actor gives out information about something to another actor)
- **Other (O)**: actors are linked another way [note taker records]

2. How are the actors linked? Please draw arrows IN BLACK showing the flow of power/level influence between the actors. Unidirectional arrows show the flow of power/influence in one direction (i.e., from actor A to actor B but not from actor B to actor A). Bidirectional arrows show that power/influence is shared between actors. Please place the appropriate abbreviation of each domain name (see print-out sheet with abbreviations) next to each arrow linking the actors. Additional type of linkages may be added during the NetMapping activities. Multiple types of linkages can be applied to the same links.
   a. Are they connected by command?
   b. Are they connected by money?
   c. Are they connected by advice?
   d. Are they connected by information?
   e. Are the connected in another way?

[The note taker takes a photo of the map.]

Power Mapping

[Building of influence towers]

The goal of this third step is to be able to identify and visualize the relative power/level of influence that actors on the map have over one another in relationship to the successful design and implementation of workplace breastfeeding interventions in Mexico.

We are interested in the relative power one actor has over another actor to influence successful workplace breastfeeding interventions with respect to the type of linkages that were defined in the previous step.

Now, please place "towers" of checker pieces on each actor card according to the influence the actors have. Please, rank each actor on a scale of 0 (does not at all influence the success of workplace breastfeeding interventions in Mexico) to 5 (influences the success of workplace breastfeeding interventions in Mexico). Please note:

The towers should be made higher for more influential actors (please place 5 checkers pieces on the card);

- The towers can be as high as you want;
- Two or more towers can be the same size;
- Actors who don't have any influence on workplace breastfeeding interventions remain at the ground level (please place 0 checkers pieces on the card).

3. How influential are the actors?
    a. How much influence do these actors have with respect to successful workplace breastfeeding interventions?
    b. From where do this influence stem?
    c. Explain the connectedness of the actors. Why do you connect them as you did?
    d. Explain the influence of the actors. Why are the towers so tall (short)?
4. Please identify the top 1-3 actors who are key in designing and implementing successful workplace breastfeeding interventions.
    a. Explain a little bit more about what makes them powerful/influential.

[The note taker takes a photo of the map. Then, remove the towers from the poster.]

Process-Influence Mapping (Process NetMap)

The goal of this last step is to identify those actors who are key to translate the policy or standard into action.

Based on the Mexican constitution and the Mexican labor law, the Mexican Secretariat of Labor and Social Welfare (STPS), the Secretariat of Health, the Mexican Social Security Institute (IMSS) and UNICEF Mexico developed a guideline for the introduction and operation of lactation rooms at the workplace.

[Show a print-out of the guideline "GUÍA PARA LA INSTALACIÓN Y FUNCIONAMIENTO DE SALAS DE LACTANCIA]

<u>Identify goals:</u>

Please place an ASTERISK on any actor card (already on the map) if the actor is involved in translating this recommendation into practice. If you identify any new actors who were not initially identified in the activities above, please add them to the map using a Post-It following the same color scheme as previously.

5. Who is involved in decision making to translate this recommendation into action?

   a. What type and level of governmental support will be needed to implement this recommendation?
   b. What type and level of other support will be needed to implement this recommendation?

Link goals:

Please draw links relating actors in this process using the RED MARKER. The links will be labeled with the numbers according to the steps in the implementation process (i.e., the first step in the process (arrow) is labeled with a 1; the second step in the process (arrow) is labeled with a 2; etc.).

6. What would the process of translating policy idea into implementation look like?
   a. Please indicate and discuss each step in the process.

Rank actors:

Next, please rank the various actors' influence on the process by placing "towers" of checker pieces according to the perceived level of influence that each institutional decision maker has. Please use the same schema for identifying higher-power decision makers by higher towers, etc., as previously done in the Power Mapping activity.

If necessary, and if time allows, ask the participant:

7. In ranking, please describe why you are ranking the decision makers as you are.

Now, please place "towers" of checker pieces on each actor card according to the influence actors have. Now, please rank each institution on a scale of 0 (does not at all influence the translation of breastfeeding policy/recommendation into action) to 5 (influences the translation of breastfeeding policy/recommendation into action) using the same tower height logic as used previously.

Final questions:

8. Consider the process above, how much time is needed to successfully implement the recommendation made in the guideline?
9. What are the biggest barriers in executing this recommendation?
10. What are the biggest facilitators in translating this recommendation into action?
11. Please identify 1-3 decision makers (actors) who are key in translating policy recommendations into action.
    a. Explain a little bit more about what makes them powerful/influential.

[The note taker takes a photo of the map.]

Conclusion

Thank you very much for participating in the NetMap interview today; we appreciate your input. We will share the results in aggregate form to all participants once the data has been analyzed by the research team.

2 Supplementary figures

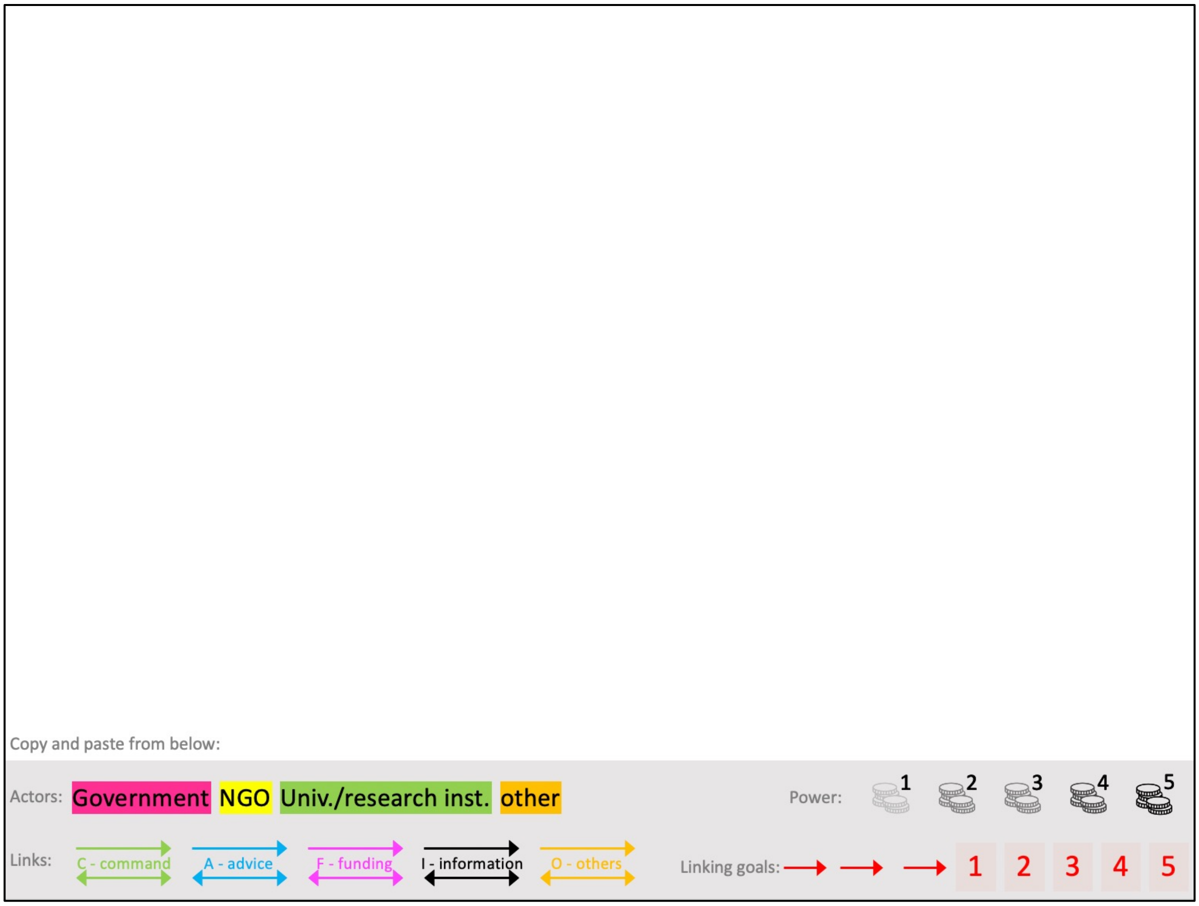

Supplementary Figure 1. Picture of the PowerPoint template used for the online NetMap interviews to identify key actors participating in the design and implementation of workplace breastfeeding interventions in Mexico.

3 Supplementary tables

Supplementary Table 1. List of all actors excluded from the analysis of actors participating in the design and implementation of workplace breastfeeding interventions in Mexico.

Actors excluded* from analysis	Actor group
National System for Integral Family Development (DIF)	Government
State offices for the National System for Integral Family Development (DIF)	Government
Federal government	Government
Institute of Legal Research at the National Autonomous University of Mexico (UNAM)	Academia
Local governments	Government
Local NGOs at state level	NGO
Mexican President	Government
United Nations Population Fund (UNFPA)	UN Organization
Yale School of Public Health	Academia

NGO: Non-governmental organization

* Exclusion criteria: Number of citations = 1 AND relative power ≤ 1

Supplementary Table 2. List of all actors, assigned actor groups, number of citations and weighted average relative power from the analysis of actors participating in the design and implementation of workplace breastfeeding interventions in Mexico.

Actor	Label	Actor group	Number of citations	Weighted average relative power
Media	1	Others	1	0.00
Labor unions	2	Others	2	0.00
National Council to Prevent Discrimination (CONAPRED)	3	Government	1	0.09
Governor of the State of Sinaloa	4	Government	1	0.09
Infancia plena	5	NGO	1	0.09
Secretariat of Labor and Employment Promotion	6	Government	1	0.09
State Secretariat of the Treasury and Public Credit	7	Government	1	0.09
Monterrey Institute of Technology and Higher Education	8	Academia	1	0.09
University of Guadalajara	9	Academia	1	0.09
UN Global Compact	10	UN Organization	1	0.09

Actor	Label	Actor group	Number of citations	Weighted average relative power
Autonomous University of the State of Hidalgo	11	Academia	1	0.09
Volunatriodo de la Secretaría de Salud (Volunteer of the Ministry of Health)	12	Others	1	0.09
Fundación DIANUI	13	NGO	1	0.18
National Institute for Perinatology	14	Government	1	0.18
National Polytechnic Institute	15	Academia	1	0.18
Mariana Villalobos	16	Others	1	0.18
Multi-stakeholder platforms (e.g., Centro Mexicano para la Filantropía)	17	Others	1	0.18
Secretariat of Public Education (SEP)	18	Government	1	0.18
State legislators	19	Government	1	0.18
Metropolitan Autonomous University	20	Academia	1	0.18
State governments	21	Government	2	0.18
World Health Organization (WHO)	22	UN Organization	2	0.18

**Actor**	**Label**	**Actor group**	**Number of citations**	**Weighted average relative power**
Private health sector	23	Others	2	0.18
Asociación Pro Lactancia Materna (APROLAM)	24	NGO	1	0.27
Chamber of Senators of the Honorable Congress of the Union	25	Government	1	0.27
National Center for Child and Adolescent Health (CeNSIA)	26	Government	1	0.27
Child daycare centers	27	Others	1	0.27
Center for Economic and Budgetary Research (CIEP)	28	Academia	1	0.27
Parliamentary Front against Hunger (FPH) of the Chamber of Deputies of the General Congress of the United Mexican States	29	NGO	1	0.27
IMSS - Bienestar	30	Government	1	0.27
Local offices of the Mexican Institute of Social Security	31	Government	1	0.27
Local offices of the National Institute for Women	32	Government	1	0.27
Punto de lactancia	33	NGO	1	0.27
Save the Children	34	NGO	1	0.27
Secretariat of Communication and Transportation	35	Government	1	0.27

Actor	Label	Actor group	Number of citations	Weighted average relative power
Secretariat of Municipal Public Services	36	Government	1	0.27
Women's NGO (e.g., GIRE)	37	NGO	1	0.27
Un Kilo de Ayuda	38	NGO	2	0.27
UN Women	39	UN Organization	2	0.27
International Labor Organization (ILO)	40	UN Organization	4	0.27
Alianza por la Salud Alimentaria	41	NGO	1	0.36
Committee on Children and Adolescent of the Chamber of Deputies	42	Government	1	0.36
Committee on Health of the Chamber of Deputies	43	Government	1	0.36
Committee on Social Security of the Chamber of Deputies	44	Government	1	0.36
Secretariat of Public Services	45	Government	1	0.36
Secretariat of National Defense	46	Government	1	0.36
Naval Secretariat	47	Government	1	0.36

Actor	Label	Actor group	Number of citations	Weighted average relative power
Judiciary	48	Government	2	0.36
National System for the Protection of Children and Adolescents (SIPINNA)	49	Government	2	0.36
National Autonomous University of Mexico (UNAM)	50	Academia	2	0.36
Doctors and Researchers in the Fight against Breast Cancer (milc)	51	NGO	2	0.36
Support groups of mothers	52	Others	1	0.45
Supreme Court of Mexico	53	Government	1	0.45
Social media	54	Others	2	0.45
Healthcare professionals	55	Others	2	0.55
Federal Commission for the Protection against Health Risks (COFEPRIS)	56	Government	3	0.55
Proyecto de Apoyo a la Lactancia Materna (PALMA)	57	NGO	3	0.55
Corporate foundations	58	Others	2	0.64
PEMEX (Mexican state-owned petroleum company)	59	Government	3	0.64
National Center for Gender Equity and Reproductive Health (CNEGSR)	60	Government	4	0.64

Actor	Label	Actor group	Number of citations	Weighted average relative power
National Institute for Women	61	Government	4	0.64
Secretariat of the Treasury and Public Credit	62	Government	2	0.73
Private companies	63	Others	2	0.73
State Secretariats of Labor and Social Welfare	64	Government	4	0.82
Families and colleagues	65	Others	2	0.91
Pan American Health Organization (PAHO)	66	UN Organization	3	0.91
Chamber of Deputies	67	Government	3	1.09
State Secretariats of Health	68	Government	5	1.09
Institute for Social Security and Services for State Workers (ISSSTE)	69	Government	6	1.09
La Leche League	70	NGO	8	1.09
Breastmilk substitute industry	71	Others	4	1.27
Association of International Board Certified Lactation Consultants in Mexico (ACCLAM)	72	NGO	8	1.27

Actor	Label	Actor group	Number of citations	Weighted average relative power
Women	73	Others	3	1.36
El Pacto por la Primera Infancia	74	NGO	7	1.55
Business groups (e.g., chamber of commerce, COPARMEX)	75	Others	6	1.73
Employers	76	Others	7	2.09
National Public Health Institute (INSP)	INSP	Academia	8	2.27
Universidad Iberoamericana Mexico City (IBERO)	IBERO	Academia	10	2.45
Federal legislators	77	Government	7	2.55
Secretariat of Health	SALUD	Government	9	2.64
United Nations Children's Fund (UNICEF)	UNICEF	UN Organization	11	3.73
Secretariat of Labor and Social Welfare	STPS	Government	11	3.82
Mexican Institute of Social Security (IMSS)	IMSS	Government	11	4.00
NGO: Non-governmental organization				
